# Identification of neuropathogenic *Varicellovirus equidalpha1* as a potential cause of respiratory disease outbreaks among horses in North Xinjiang, China, from 2021-2023

**DOI:** 10.1186/s12917-024-03925-z

**Published:** 2024-02-27

**Authors:** Panpan Tong, Enhui Yang, Bin Liu, Shuyao tian, Youtu Suo, JuanJuan Pan, Yueyi Dang, Nuerlan Palidan, Chenyang Jia, Ling Kuang, Jinxin Xie

**Affiliations:** 1 Laboratory of Animal Etiology and Epidemiology, College of Veterinary Medicine, Xinjiang Agricultural University, Urumqi, 830052 China; 2 Hengxing Equestrian Club, Changji, 831100 China; 3 Zhaosu Horse Barn in Yili, Zhaosu, 835602 China; 4 Vocational Technical School of Zhaosu, Zhaosu, 835600 China; 5 Xinjiang Nucleic Acid Testing Center, Xinjiang Medical University, Urumqi, 830011 China

**Keywords:** Horses, Respiratory disease, *Varicellovirus equidalpha1*, ORF33 gene, ORF30 gene, Neuropathogenicity

## Abstract

**Background:**

*Varicellovirus equidalpha1* (formerly Equid alphaherpesvirus 1, EqAHV-1) is among the most important viruses responsible for respiratory disease outbreaks among horses throughout the world. No reports to date have detailed the association between EqAHV-1 and respiratory disease among horses in China. This study described one such outbreak among a population of horses in north Xinjiang that occurred from April 2021 - May 2023.

**Results:**

qPCR revealed that EqAHV-1 was detectable in all samples and this virus was identified as a possible source of respiratory disease, although a limited subset of these samples were also positive for EqAHV-2, EqAHV-4, and EqAHV-5. In total, three EqAHV-1 strains responsible for causing respiratory illness in horses were isolated successfully, and full-length ORF33 sequence comparisonsand phylogenetic analyses indicated that these isolates may have originated from EqAHV-1 strains detected in Yili horse abortions. ORF30 sequence data additionally suggested that these strains were neuropathic, as evidenced by the presence of a guanine residue at nucleotide position 2254 corresponding to the aspartic acid present at position 752 in the DNA polymerase encoded by this virus.

**Conclusion:**

This study is the first report of an outbreak of respiratory disease among horses in China caused by EqAHV-1. ORF30 sequence characterization revealed that these EqAHV-1 strains harbored a neuropathogenic genotype. Given the detection of this virus in horses suffering from respiratory disease, concern is warranted with respect to this neuropathogenic EqAHV-1 outbreak.

**Supplementary Information:**

The online version contains supplementary material available at 10.1186/s12917-024-03925-z.

## Background

*Varicellovirus equidalpha1* formerly know as Equid alphaherpesvirus 1 is an *Alphaherpesvirinae* subfamily member of the *Varicellovirus* genus that represents an important cause of equine disease, frequently causing abortion, respiratory illness, neurological disease, and ocular disease [1–20]. Given the potential severity of these symptoms, EqAHV-1 thus represents an important threat to the health of individual horses and to the global equine industry as a whole [2–20]. The EqAHV-1 genome is 150 kbp in length, encoding as many as 80 open reading frames (ORFs) [20–22]. ORF30 codes for the viral DNA polymerase, and the nucleotide present at position 2254 (A/G/C) is strongly, albeit not exclusively, associated with whether or not the EqAHV-1 strain is neuropathogenic [6–19]. ORF33 encodes the highly conserved envelope glycoprotein B (gB), which is often targeted when detecting EqAHV-1 [6, 16, 20–22].

An outbreak of acute respiratory disease with an incubation period of under 24 h was detected among Yili mares, thoroughbred stallions, and thoroughbred racehorses at the Hengxing Equestrian Club in Changji, northern Xinjiang, China in April 2021. This respiratory disease has since spread rapidly through breeding mares on other farms in cities throughout northern Xinjiang including Changji, Kelamayi, Shihezi, and Urumqi. An outbreak of acute respiratory illness additionally occurred during horse racing in Zhaosu County, Yining in northern Xinjiang in August 2022. In May of 2023, this same respiratory disease again spread among horses in Zhaosu County during breeding season. The morbidity rate of the respiratory diaease ranges from 50 to 70%. Symptoms of this disease reportedly include pyrexia (> 40 °C), cough, purulent nasal discharge, reduced appetite, and weight loss, but lack neurological signs. Following veterinary isolation in new premises, sick horses are treated with both intramuscular (Sulfamethoxydiazine Sodium and Astragalus polysaccharides) and intravenous (Sodium salicylate, Urotropine, and Calcium gluconate) injections, all horses recovered from this illness. At present, horses in Xinjiang do not undergo any routinely scheduled vaccination.

In the present study, there was no evidence that these horses were infected with the H3N8 equine influenza virus, which has been reported as a cause of respiratory disease in horses in China [23]. To identify viruses potentially responsible for these outbreaks of respiratory disease, nasal swabs from the majority of sick horses were collected and analyzed via PCR or qPCR to detect EqAHV-1, -4, -2, and − 5 in these samples. The ORF30 and ORF33 sequences from identified EqAHV-1 isolates were then subject to further characterization. This study is the first to have been conducted with a focus on EqAHV-1 isolates responsible for equine respiratory disease in China, will strongly promote EqAHV-1 vaccine development.

## Materials and methods

### Sample collection

In April 2021, August 2022, and May 2023, 162 total nasal swabs were collected from symptomatic horses during respiratory disease outbreaks in Changji (*n* = 67), Shihezi (*n* = 12), Kelamayi (*n* = 11), Urumqi (*n* = 48), and Yining (*n* = 24) in north Xinjiang, China. An additional 416 nasal swabs were collected from clinically healthy control horses. Each of these sample was collected, and placed in a tube containing 1.5 mL of phosphate buffer and stored at -80 °C by the farm’s veterinarian according to the approved procedures.

### EqAHV detection

Nasal swab samples were vortexed, centrifuged, and a 200 µL supernatant sample was used to extract viral nucleic acids using a kit (Geneaid Biotech Co.) based on provided instructions. The presence of EqAHV-1 DNA in these samples was further confirmed through a TaqMan-MGB qPCR assay targeting a portion of ORF68 using the primers and probes listed in Table 1. Target genes were amplified in qPCR assays using SuperReal PreMix (Tiangen Biotech Co.) with the following thermocycler settings: 95 °C for 15 min; 45 cycles of 94 °C 30 s and 60 °C 30 s. EqAHV-2 (716 nt), EqAHV-4 (587 nt), and EqAHV-5 (881 nt) DNA was additionally detected in these samples via PCR using primers targeting portions of ORF33 (EqAHV-4) or ORF8 (EqAHV-5 or EqAHV-2) encoding the gB in Table 1.

**Table 1:** Primers and probe for EqAHVs in this study

Primer	Sequence (5´-3´)	Product size (bp)	Reference
EqAHV-1 Forward Primer	AATACCATGGACCACCGTTGA	58	
EqAHV-1 Reverse Primer	TCCACCCGCCGCTCTT		
EqAHV-1-Probe	FAM-CATAATCATCCGCTCAAAT-MGB		
EqAHV-1 ORF33 Forward Primer1	ATGTCCTCTGGTTGCCGTTCTG	1644	[16]
EqAHV-1 ORF33 Reverse Primer1	CTCGTTTGTGCAAGCGATGTAG		
EqAHV-1 ORF33 Forward Primer2	GTGAGATCTAACCGCACCTACGAC	1463	[16]
EqAHV-1 ORF33 Reverse Primer2	TTAAACCATTTTTTCATYTTCCATG		
EqAHV-1 ORF30 Forward Primer	CGGAGTAAGGCTTGTGGTTTCG	559	[7, 16]
EqAHV-1 ORF30 Reverse Primer	GTGGGCTACCAGGGAGCAAAG		
EqAHV-4 ORF33 Forward Primer	GGGTCCTACAGATTTACTATTCG	587	[7]
EqAHV-4 ORF33 Reverse Primer	ACACCACGCAGTTGCTATCCTAC		
EqAHV-5 ORF8 Forward Primer	TGTATCACCGTGGACCAAGAGAG	881	[7, 24]
EqAHV-5 ORF8 Reverse Primer	TCAAAGATGGATCTTCTCTGAGTG		
EqAHV-2 ORF8 Forward Primer	GGTGACACTATAGAGATGTCRCC	716	[7, 24]
EqAHV-2 ORF8 Reverse Primer	CTGTTGATGCTCTTTCTGAGATTG		

### Isolation, screening, and electron microscopy of EqAHV-1

MDBK cells were used for the in vitro isolation of EqAHV-1 as reported previously [16]. Briefly, PCR-positive samples were centrifuged for 3 min at 12,000 x g, after which supernatants were passed through a 0.22 μm filter, used to inoculate MDBK cells for 2 h at 37 °C in a 5% CO_2_ incubator, and the inoculum was subsequently discarded and replaced with DMEM supplemented with 2% fetal bovine serum (FBS). Cells were then maintained for 72 h, followed by three rounds of freezing and thawing to harvest viruses, followed by repeated inoculation for six cell passages. Cytopathic effect (CPE) testing was then performed daily following inoculation, and the full-length OFR33 and partial ORF30 sequences of EqAHV-1 were amplified using the primers provided in Table 1. Positive PCR amplicons were then inserted into the pESI-T vector (Yeasen Biotech) and used for the transformation of *E. coli* DH5α Chemically Competent Cell (Weidi Biotech), after which three clones per amplicon were selected for Sanger sequencing (Sangon Biotech). For electron microscopy, EqAHV-1 particles purified by sucrose density gradient centrifugation as previously described [16] were negatively stained with 2% phosphotungstic acid.

### Multiple sequence alignment and phylogenetic analyses

**Fig. 1:**
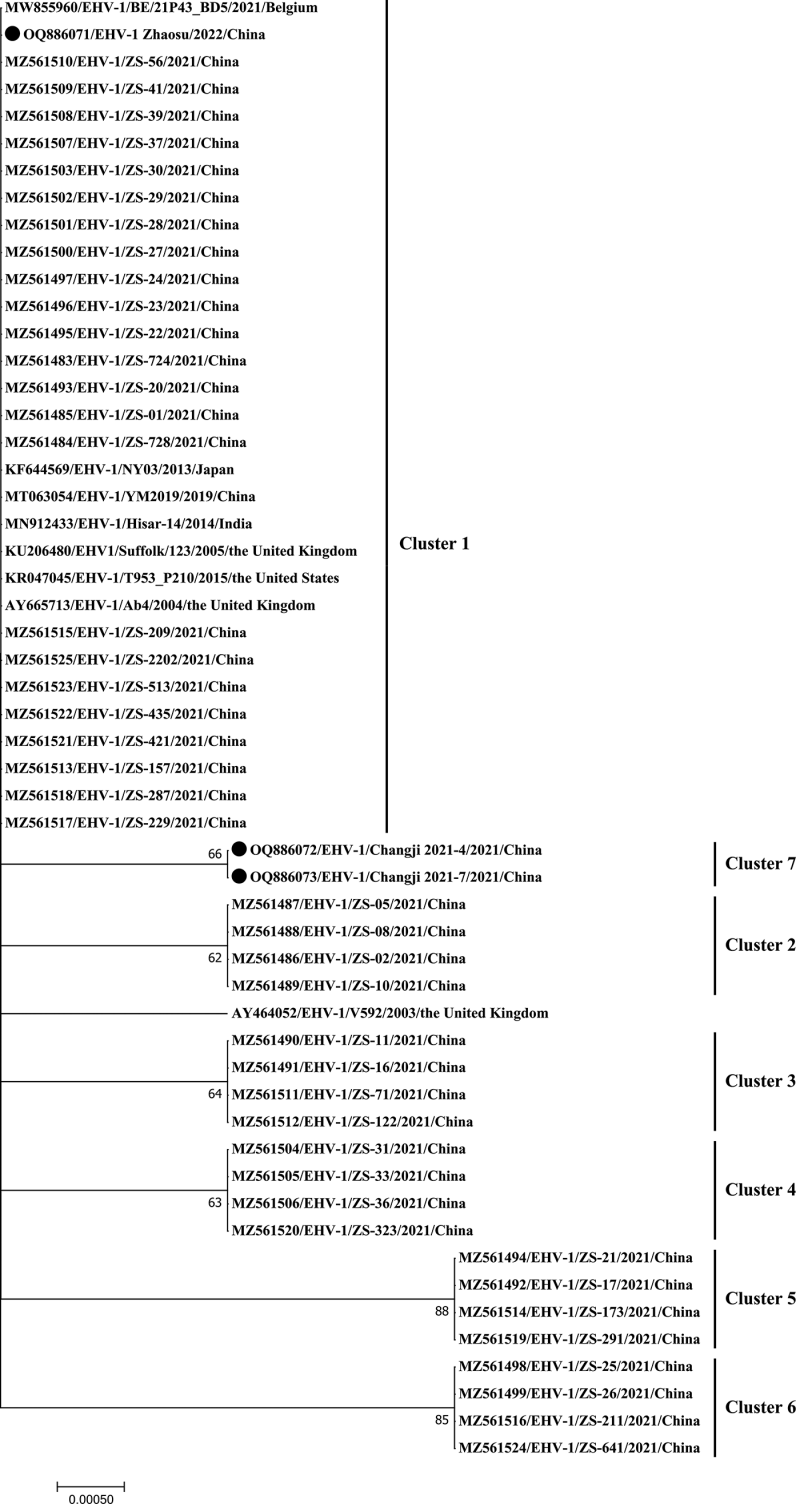
A maximum-likelihood phylogenic network developed based on EqAHV-1 ORF33 gene sequences and the Tamura–Nei model (MEGA7). EqAHV-1 strains represented in the present study are represented with black circles

Additional information, including the GenBank accession numbers, for the sequences in this study is provided in Fig. 1. All full-length ORF33 and partial ORF30 EqAHV-1 nucleotide sequences isolated in this study were submitted to GenBank, with the following accession numbers: OQ886071-OQ886076. The MegAlign software was used to analyze these sequences in Lasergene v7.1, and a maximum-likelihood phylogenetic network of all target sequences was constructed in MEGA7 with the Tamura-Nei model. Tree topological accuracy was assessed with 1,000 bootstrap replicates [25].

## Results

### EqAHV detection

Initial PCR testing confirmed that EqAHV-1, -2, -4, and − 5 were detectable in nasal swabs from horses suffering from respiratory disease, with respective positivity rates of 100% (162/162), 13.6% (22/162), 9.3% (15/162), and 9.3% (15/162). In contrast, the positivity rates in nasal swabs from healthy horses were 0% (0/416), 11.1% (46/416), 5.1% (21/416), and 16.6% (69/416), respectively. Seven sick horses were co-infected with all four of these EqAHV varieties, while three healthy horses were positive for co-infection with EqAHV-2, EqAHV-4, and EqAHV-5. These results thus suggested that EqAHV-1 was the potential causative pathogen responsible for these outbreaks of respiratory disease among horses in northern Xinjiang, China.

### EqAHV-1 isolation

Next, viral isolation was performed using three nasal swabs collected from symptomatic horses that were only positive for EqAHV-1. Using MDBK cells as recipients, all three of these viral isolates exhibited clear CPEs by passage six, and PCR subsequently confirmed the successful isolation of three EqAHV-1 isolates (Zhaosu/2022, Changji/2021-4, and Changji/2021-7) after eight passages. Relative to control cells that underwent mock infection, MDBK cells that were infected with EqAHV-1 exhibited rounding, clustering, fusion, shedding, and void formation at 24 h post-infection. Transmission electron microscopy revealed that the viral particles in the EqAHV-1 Zhaosu/2022 isolate exhibited the typical morphological characteristics of EqAHV-1, with virions ~200 nm in diameter.

### ORF33 and ORF30 sequences analyses

The full-length (2793 nt) ORF33 sequences from these three EqAHV-1 isolates were next compared, revealing respective nucleotide and amino acid similarity levels of 99.9–100% and 99.8–100%, (accession numbers: OQ886071-OQ886073), confirming the high degree of evolutionary conservation of this gene. These sequences were additionally compared to EqAHV-1 reference strains from China (accession numbers: MT063054, and MZ561483-MZ561525), the United Kingdom (V592: accession number. AY464052, Ab4: accession number. AY665713, and Suffolk/123/2005: accession number. KU206480), Japan (NY03: accession number. KF644569), the USA (T953_P210/2015: accession number. KR047045), India (Hisar-14/2014: accession number. MN912433), and Belgium (BE/21P43_BD5: accession number. MW855960). These results revealed DNA and amino acid sequence similarity levels ranging from 99.5 to 100% and 99–100% (Table 2), respectively. A phylogenetic network was then constructed based on these sequences, revealing the classification of the EqAHV-1 Zhaosu/2022 strain in one of six known clades [7], whereas EqAHV-1 isolates Changji/2021-4 and Changji/2021-7 formed a novel clade that was designated as cluster seven (Fig. 1).

**Table 2:** Nucleotide (upper right) and amino acid (bottom left) similarity of the ORF33 sequence between EqAHV-1 identified in this study and EqAHV-1 reference strains

	3 EHV-1 isolates in this study	43 EHV-1 detected in Yili horse abortion	EHV-1 Ab4	EHV-1 NY03	EHV-1 T953_P210	EHV-1 Suffolk/123	EHV-1 Hisar-14	EHV-1 YM2019	EHV-1 BE/21P43_BD5	EHV-1 V592
**3 EHV-1 isolates** **in this study**		99.5–100	100	100	99.9	99.9	99.8	100	99.8	99.9
**43 EHV-1 detected in** **Yili horse abortion**	99–100		99.7–100	99.7–100	99.7–100	99.7–100	99.7–100	99.7–100	99.5–99.9	99.5–99.9
**EHV-1** **Ab4**	99.9	99.5–100		99.9	99.9	99.9	99.8	100	99.9	99.9
**EHV-1** **NY03**	99.9	99.5–100	100		99.9	99.9	99.8	100	99.9	99.9
**EHV-1** **T953_P210**	99.6	99.5–100	99.7	99.7		99.9	99.9	99.9	99.9	99.9
**EHV-1** **Suffolk/123**	99.8	99.5–100	99.9	99.9	99.8		99.8	99.9	99.9	99.9
**EHV-1** **Hisar-14**	99.7	99.5–100	99.8	99.8	99.9	99.9		99.8	99.8	99.9
**EHV-1** **YM2019**	99.9	99.5–100	100	100	99.7	99.9	99.8		99.9	99.9
**EHV-1** **BE/21P43_BD5**	99.6	99.0-99.5	99.7	99.7	99.8	99.8	99.9	99.7		99.9
**EHV-1** **V592**	99.7	99.5–100	99.8	99.8	99.9	99.9	100	99.8	99.9	

**Fig. 2:**
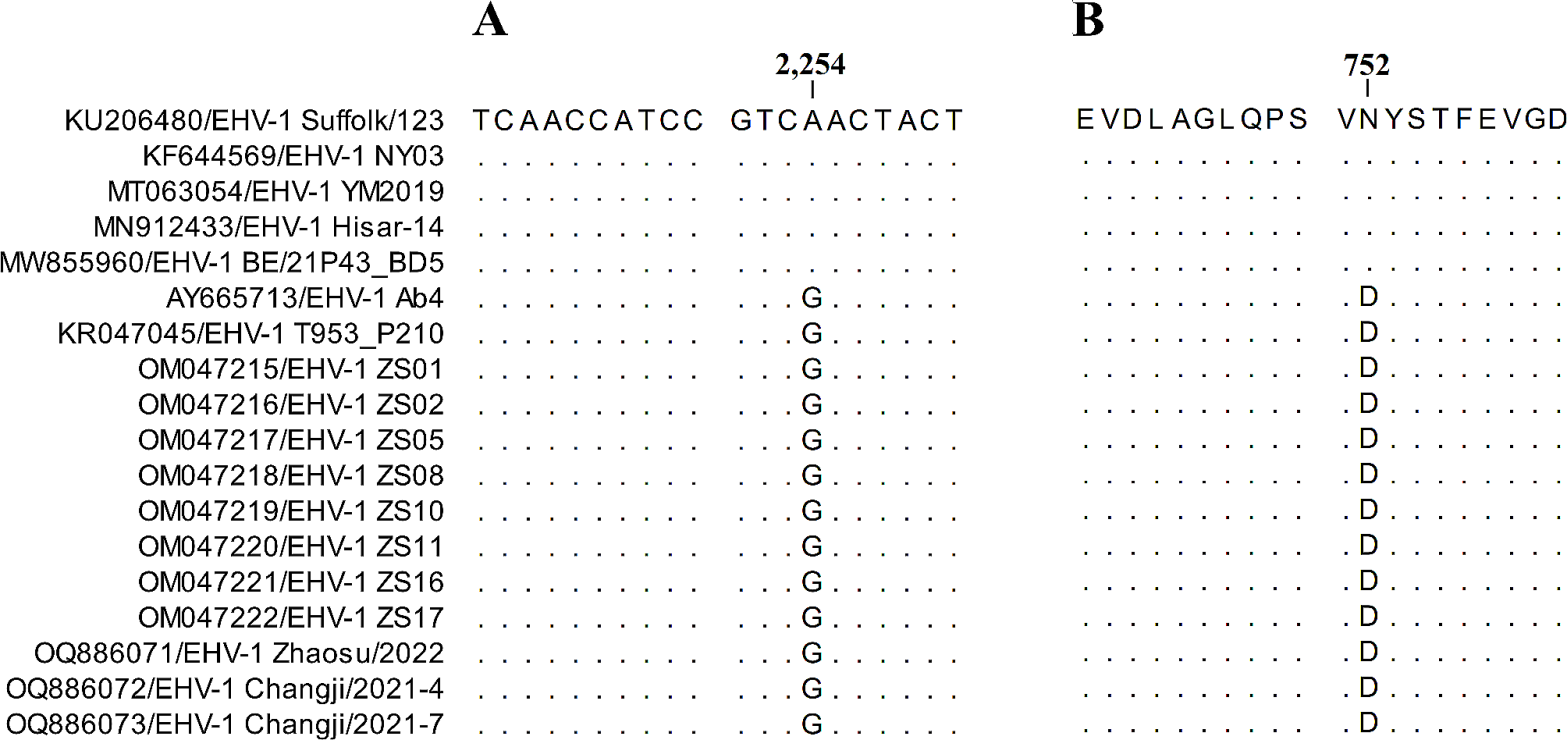
ORF30 gene sequence analyses. (**A**) The G2254 polymorphism detected in all EqAHV-1 strains from this study. (**B**) The D752 polymorphism detected in all EqAHV-1 strains from this study. Dots represent the sequence identity when aligned with the EqAHV-1 Suffolk/123 isolate (CLC Sequence Viewer 8)

To further explore the potential neuropathogenicity of these three EqAHV-1 isolates from horses with respiratory disease in China, a 559 nucleotide fragment of the EqAHV-1 ORF30 was next amplified using previously reported primers [7, 16]. These three partial ORF30 sequences exhibited 100% nucleotide and amino acid sequence identity (accession numbers: OQ886074-OQ886076), consistent with the high degree of genetic conservation. Consistent with the ZS01, ZS02, ZS05, ZS08, ZS10, ZS11, ZS16, ZS17, Ab4, and T953_P210 neuropathogenic EqAHV-1 reference strains, all three of these EqAHV-1 isolates harbored a G at position 2254 (D in position 752 of the viral DNA polymerase) (Fig. 2), suggesting that these strains represent neuropathogenic EqAHV-1 isolates.

## Discussion

Outbreaks of respiratory disease have a substantial negative impact on the global equine industry, resulting in the disruption of stallion mating, impaired training, the need for horses to bow out of competitions, and the high costs associated with quarantine/isolation and the treatment of infected horses [8–13]. Symptoms of the sick horses analyzed in this study included pyrexia, cough, purulent nasal discharge, reduced appetite, and weight loss, disrupting horse breeding, trading, training, and racing, thereby resulting in substantial economic losses for the equine industry in Xinjiang.

Multiple prior reports have identified equine influenza virus (EIV), EqAHV-1, EqAHV-4, equine rhinitis viruses A (ERAV) and B (ERBV), and equine arteritis virus (EAV) as the most common causes of respiratory illness among horses [11–13]. H3N8 EIV has previously been identified as being associated with respiratory illness among Chinese horses [23], but this virus was undetectable in nasal swabs from horses in north Xinjiang during the 2021–2023 outbreak discussed herein. Another recent publication focused on Xinjiang detected EqAHV-4, -2, and − 5 in nasal swabs from thoroughbred foals exhibiting respiratory symptoms [24]. In the present study, EqAHV-4, -2, and − 5 were respectively detected in 9.4%, 13.8%, and 9.4% of nasal swabs from horses with respiratory symptoms, but they were also detected in 5.1%, 11.1%, and 16.6% of nasal swabs from healthy horses, thus suggesting that these three viruses were not responsible for this outbreak of equine respiratory disease.

EqAHV-1 is a viral pathogen that has previously been demonstrated to cause respiratory illness, abortion, and stillbirth among horses [1–20]. However, there have only been four publications from 2016 to 2022 indicating the presence of EqAHV-1 in aborted fetal lung tissue samples from mares in China. Three of these studies determined that EqAHV-1 was responsible for Yili horse abortions at the Chinese State Studs in Zhaosu County in 2015 [5] and 2021 [7, 16], while one detected this virus in aborted tissue samples from a Przewalski’s horse conservation center in Jimusaer County in 2019 [17]. No studies to date have documented a role for EqAHV-1 as a cause of respiratory disease among horses in China. The present qPCR data, however, confirmed that 100% of nasal swabs from symptomatic horses suffering from respiratory disease in the analyzed outbreaks were positive for EqAHV-1, whereas 0% of samples from healthy horses were positive for this virus.

These results suggested that EqAHV-1 is a strong candidate as the pathogen responsible for the high rates of respiratory disease among the horse population in north Xinjiang from 2021 to 2023. This study was the first report of the detection of EqAHV-1 in nasal swabs from horses in China, supporting the tentative identification of this virus as the cause of equine respiratory illness. Future molecular and epidemiological studies will be conducted with the goal of more broadly evaluating the relationship between EqAHV-1 and respiratory disease among the Chinese horse population. The EqAHV-1 isolates detected in the present study and a previous report [16] will additionally be leveraged by our team in an effort to design an attenuated EqAHV-1 vaccine that will be tested for its ability to prevent respiratory disease and abortion incidence among horses in China.

An outbreak of abortions caused by EqAHV-1 was detected among Yili mares at the Chinese State Studs in Zhaosu County in January 2021. The first clusters of respiratory disease reported in the present study occurred in April 2021 among Yili mares, thoroughbred stallions, and thoroughbred racehorses, followed by a second event in August 2022 during Yili horse racing, and a third outbreak of acute respiratory disease among Yili mares in May 2023. Subsequent epidemiological analyses suggested that the transportation of Yili horses across regions for the purposes of racing and breeding contributed to the spread of disease among farms in northern Xinjiang from 2021 to 2023.

Further comparisons of the full-length ORF33 sequences from the three EqAHV-1 isolates in the present study and reference EqAHV-1 strains revealed a high degree of similarity (99.5–100% DNA sequence similarity, 99–100% amino acid sequence similarity) among strains associated with disease in Yili horses (Table 2). The EqAHV-1 Zhaosu/2022 strain isolated in this study was clustered into one of six different clades constructed based on all EqAHV-1 sequences detected in Yili mare storm. Overall, these results suggested that the EqAHV-1 isolates responsible for the more recent outbreaks of respiratory disease may have originated from EqAHV-1 strains responsible for the incidence of abortion among Yili horses.

In prior studies, the nucleotide at position 2254 in ORF30 (A/G/C) has been closely tied to the incidence of neurological disease in animals infected with EqAHV-1 [6–19]. Most viral samples isolated from aborted fetuses, for example, are non-neuropathogenic strains harboring an A at this position which translates to an asparagine (N) at position 752 in the final protein [13]. Prior to 2019, as reported by Yang et al. [5] who detected the EqAHV-1-XJ2015 strain in aborted fetal lung tissue samples isolated from Yili horses and Hu et al. [17] who identified the YM2019 strain in aborted fetal lung tissue samples from Przewalski’s horses, these A2254 strains are then only EqAHV-1 isolates reported in aborted horses in China [15]. However, a report published by Tong et al. [6] identified G2254 strains that were associated with high rates of abortion among Yili horses in north Xinjiang in 2021. In line with their report, the EqAHV-1 isolates associated with equine respiratory disease in north Xinjiang in the present study harbored a G at position 2254 (D in position 752), and these isolates were thus classified as a neuropathogenic EqAHV-1 strain. These results thus strongly suggest that neuropathogenic EqAHV-1 can cause both abortions and respiratory disease among horses in China.

Many reports have previously noted that relative to neuropathogenic EqAHV-1 strains (G2254), non-neuropathogenic strains (A2254) are more closely associated with respiratory disease in horses [14, 18]. Four recent reports published in the USA and Europe identified a novel ORF30 EqAHV-1 DNA polymerase genotype (C2254/H752) closely associated with respiratory illness [9, 12, 13, 15]. In contrast, a neuropathogenic strain was isolated from horses with respiratory ailments in this study. Future reports will seek to explore the incidence of infections caused by the A2254 and C2254 genotypes of EqAHV-1 among horses in China with respiratory disease.

## Conclusion

In conclusion, EqAHV-1 was herein detected in samples collected from horses suffering from respiratory symptoms such that it was identified as the likely cause of an outbreak of equine respiratory disease that occurred in north Xinjiang from 2021 to 2023. Subsequent characterization of these EqAHV-1 isolates suggested that the neuropathogenic EqAHV-1 isolates initially identified as drivers of respiratory disease may have also contributed to an increase in abortions affecting Yili horses. These results are expected to raise awareness regarding the potential for neuropathogenic EqAHV-1 to contribute to respiratory illness among horses in China, spurring efforts to design a vaccine that can reliably combat the spread and severity of the associated disease.

### Electronic supplementary material

Below is the link to the electronic supplementary material.

Supplementary Material 1

## Data Availability

All data generated or analyzed during this study are included in this published article and its additional files. Sequences of EqAHV-1 isolates in this study have been submitted to GenBank under accession numbers: OQ886071-OQ886076.

## Abbreviations

EqAHV: *Varicellovirus equidalpha1*
ORF: Open reading frame
G: Guanidine
A: Adenine
N: Asparagine
D: Aspartic acid
C: Cytosine
H: Histidine
