# Supplementary material for: Identification of neuropathogenic Varicellovirus equidalpha1 as a potential cause of respiratory disease outbreaks among horses in North Xinjiang, China, from 2021-2023
Source: BMC Vet Res. 2024 Feb 27;20:77. doi: 10.1186/s12917-024-03925-z (PMC10898184; doi:10.1186/s12917-024-03925-z)
Supplement: Supplementary file 1 — Supplementary Material 1 [file 12917_2024_3925_MOESM1_ESM.docx]

EHV-1 Kelamayi/2021-1:

GTGGGCTACCAGGGAGCAAAGGTTCTAGACCCCGTATCCGGCTTTCATGTGGACCCCGTGGTTGTGTTTGACTTCGCTAGCTTATACCCAAGCATTATCCAGGCCCATAACCTCTGTTTCACCACCCTGGCGCTCGATGAAGTGGATCTGGCCGGGCTTCAACCATCCGTCGACTACTCGACGTTCGAGGTGGGTGACCAAAAGTTATTTTTTGTCCACGCCCATATTCGCGAAAGCCTGCTTGGCATCTTGCTGCGCGACTGGCTGGCCATGCGAAAGGCGGTGAGGGCGCGAATCCCCACCAGCACCCCCGAGGAGGCAGTTTTACTAGATAAGCAGCAGTCTGCGATTAAGGTGATATGCAACTCGGTTTACGGATTCACGGGGGTGGCAAACGGCCTGTTGCCGTGTCTGAGGATAGCGGCTACCGTTACCACGATAGGACGCGACATGCTCCTCAAGACCCGAGATTACGTTCACTCTCGTTGGGCGACGCGCGAGCTGCTGGAGGACAATTTTCCAGGGGCTATAGGTTTCCGAAACCACAAGCCTTACTCCG

EHV-1 Shihezi/2021-3:

GTGGGCTACCAGGGAGCAAAGGTTCTAGACCCCGTATCCGGCTTTCATGTGGACCCCGTGGTTGTGTTTGACTTCGCTAGCTTATACCCAAGCATTATCCAGGCCCATAACCTCTGTTTCACCACCCTGGCGCTCGATGAAGTGGATCTGGCCGGGCTTCAACCATCCGTCGACTACTCGACGTTCGAGGTGGGTGACCAAAAGTTATTTTTTGTCCACGCCCATATTCGCGAAAGCCTGCTTGGCATCTTGCTGCGCGACTGGCTGGCCATGCGAAAGGCGGTGAGGGCGCGAATCCCCACCAGCACCCCCGAGGAGGCAGTTTTACTAGATAAGCAGCAGTCTGCGATTAAGGTGATATGCAACTCGGTTTACGGATTCACGGGGGTGGCAAACGGCCTGTTGCCGTGTCTGAGGATAGCGGCTACCGTTACCACGATAGGACGCGACATGCTCCTCAAGACCCGAGATTACGTTCACTCTCGTTGGGCGACGCGCGAGCTGCTGGAGGACAATTTTCCAGGGGCTATAGGTTTCCGAAACCACAAGCCTTACTCCG

EHV-1 Shihezi/2021-7:

GTGGGCTACCAGGGAGCAAAGGTTCTAGACCCCGTATCCGGCTTTCATGTGGACCCCGTGGTTGTGTTTGACTTCGCTAGCTTATACCCAAGCATTATCCAGGCCCATAACCTCTGTTTCACCACCCTGGCGCTCGATGAAGTGGATCTGGCCGGGCTTCAACCATCCGTCGACTACTCGACGTTCGAGGTGGGTGACCAAAAGTTATTTTTTGTCCACGCCCATATTCGCGAAAGCCTGCTTGGCATCTTGCTGCGCGACTGGCTGGCCATGCGAAAGGCGGTGAGGGCGCGAATCCCCACCAGCACCCCCGAGGAGGCAGTTTTACTAGATAAGCAGCAGTCTGCGATTAAGGTGATATGCAACTCGGTTTACGGATTCACGGGGGTGGCAAACGGCCTGTTGCCGTGTCTGAGGATAGCGGCTACCGTTACCACGATAGGACGCGACATGCTCCTCAAGACCCGAGATTACGTTCACTCTCGTTGGGCGACGCGCGAGCTGCTGGAGGACAATTTTCCAGGGGCTATAGGTTTCCGAAACCACAAGCCTTACTCCG

EHV-1 Shihezi/2021-9:

GTGGGCTACCAGGGAGCAAAGGTTCTAGACCCCGTATCCGGCTTTCATGTGGACCCCGTGGTTGTGTTTGACTTCGCTAGCTTATACCCAAGCATTATCCAGGCCCATAACCTCTGTTTCACCACCCTGGCGCTCGATGAAGTGGATCTGGCCGGGCTTCAACCATCCGTCGACTACTCGACGTTCGAGGTGGGTGACCAAAAGTTATTTTTTGTCCACGCCCATATTCGCGAAAGCCTGCTTGGCATCTTGCTGCGCGACTGGCTGGCCATGCGAAAGGCGGTGAGGGCGCGAATCCCCACCAGCACCCCCGAGGAGGCAGTTTTACTAGATAAGCAGCAGTCTGCGATTAAGGTGATATGCAACTCGGTTTACGGATTCACGGGGGTGGCAAACGGCCTGTTGCCGTGTCTGAGGATAGCGGCTACCGTTACCACGATAGGACGCGACATGCTCCTCAAGACCCGAGATTACGTTCACTCTCGTTGGGCGACGCGCGAGCTGCTGGAGGACAATTTTCCAGGGGCTATAGGTTTCCGAAACCACAAGCCTTACTCCG

EHV-1 Urumqi/2021-1:

GTGGGCTACCAGGGAGCAAAGGTTCTAGACCCCGTATCCGGCTTTCATGTGGACCCCGTGGTTGTGTTTGACTTCGCTAGCTTATACCCAAGCATTATCCAGGCCCATAACCTCTGTTTCACCACCCTGGCGCTCGATGAAGTGGATCTGGCCGGGCTTCAACCATCCGTCGACTACTCGACGTTCGAGGTGGGTGACCAAAAGTTATTTTTTGTCCACGCCCATATTCGCGAAAGCCTGCTTGGCATCTTGCTGCGCGACTGGCTGGCCATGCGAAAGGCGGTGAGGGCGCGAATCCCCACCAGCACCCCCGAGGAGGCAGTTTTACTAGATAAGCAGCAGTCTGCGATTAAGGTGATATGCAACTCGGTTTACGGATTCACGGGGGTGGCAAACGGCCTGTTGCCGTGTCTGAGGATAGCGGCTACCGTTACCACGATAGGACGCGACATGCTCCTCAAGACCCGAGATTACGTTCACTCTCGTTGGGCGACGCGCGAGCTGCTGGAGGACAATTTTCCAGGGGCTATAGGTTTCCGAAACCACAAGCCTTACTCCG

EHV-1 Urumqi/2021-16:

GTGGGCTACCAGGGAGCAAAGGTTCTAGACCCCGTATCCGGCTTTCATGTGGACCCCGTGGTTGTGTTTGACTTCGCTAGCTTATACCCAAGCATTATCCAGGCCCATAACCTCTGTTTCACCACCCTGGCGCTCGATGAAGTGGATCTGGCCGGGCTTCAACCATCCGTCGACTACTCGACGTTCGAGGTGGGTGACCAAAAGTTATTTTTTGTCCACGCCCATATTCGCGAAAGCCTGCTTGGCATCTTGCTGCGCGACTGGCTGGCCATGCGAAAGGCGGTGAGGGCGCGAATCCCCACCAGCACCCCCGAGGAGGCAGTTTTACTAGATAAGCAGCAGTCTGCGATTAAGGTGATATGCAACTCGGTTTACGGATTCACGGGGGTGGCAAACGGCCTGTTGCCGTGTCTGAGGATAGCGGCTACCGTTACCACGATAGGACGCGACATGCTCCTCAAGACCCGAGATTACGTTCACTCTCGTTGGGCGACGCGCGAGCTGCTGGAGGACAATTTTCCAGGGGCTATAGGTTTCCGAAACCACAAGCCTTACTCCG

EHV-1 Urumqi/2021-23:

GTGGGCTACCAGGGAGCAAAGGTTCTAGACCCCGTATCCGGCTTTCATGTGGACCCCGTGGTTGTGTTTGACTTCGCTAGCTTATACCCAAGCATTATCCAGGCCCATAACCTCTGTTTCACCACCCTGGCGCTCGATGAAGTGGATCTGGCCGGGCTTCAACCATCCGTCGACTACTCGACGTTCGAGGTGGGTGACCAAAAGTTATTTTTTGTCCACGCCCATATTCGCGAAAGCCTGCTTGGCATCTTGCTGCGCGACTGGCTGGCCATGCGAAAGGCGGTGAGGGCGCGAATCCCCACCAGCACCCCCGAGGAGGCAGTTTTACTAGATAAGCAGCAGTCTGCGATTAAGGTGATATGCAACTCGGTTTACGGATTCACGGGGGTGGCAAACGGCCTGTTGCCGTGTCTGAGGATAGCGGCTACCGTTACCACGATAGGACGCGACATGCTCCTCAAGACCCGAGATTACGTTCACTCTCGTTGGGCGACGCGCGAGCTGCTGGAGGACAATTTTCCAGGGGCTATAGGTTTCCGAAACCACAAGCCTTACTCCG
